# Integrated botanical and cultural strategies for management of major insect pests in maize

**DOI:** 10.1093/jisesa/ieag060

**Published:** 2026-06-27

**Authors:** Kassahun Yimenu Abera, Getnet Atenafu Abate, Melkamsew Berihun Geremew

**Affiliations:** Department of Horticulture, College of Agriculture and Environmental Sciences, Debre Markos University, Debre Markos, Ethiopia; Department of Biology, College of Natural and Computational Sciences, Debre Markos University, Debre Markos, Ethiopia; Department of Horticulture, College of Agriculture and Environmental Sciences, Debre Markos University, Debre Markos, Ethiopia

**Keywords:** maize pest management, botanical insecticides, integrated pest management, sustainable agriculture

## Abstract

Maize (*Zea mays* L.) is a staple crop for millions of people worldwide, yet its productivity is constantly under threat from pests such as fall armyworm (*Spodoptera frugiperda* J.E. Smith), stem borers (*Busseola fusca* Fuller and *Chilo partellus* Swinhoe), maize aphids (*Rhopalosiphum maidis* Fitch), and maize weevil (*Sitophilus zeamais* Motschulsky). These pests can cause devastating losses of 20–50% in the field and up to 40% during storage. The pests are increasingly resistant to chemical insecticides, and there are growing concerns about environmental and health impacts. Sustainable alternatives are urgently needed. This review brings together insights from 34 studies (2000–2025) on plant-based insecticides, cultural practices, and their integration for maize pest management. Across the selected studies, larval mortality due to botanical extracts ranged from 45% to 92%, depending on the plant species used, formulation, and target pest. These values reflect the range reported in different studies rather than results from a formal meta-analysis. Cultural practices like intercropping, crop rotation, adjusting planting dates, and field sanitation lowered pest pressure by 18–80% and boosted yields by 8–45%. Combining these approaches was even more powerful, reducing fall armyworm damage by 65–88%, increasing yields up to 52%, and supporting beneficial insects. Despite these successes, challenges remain, especially in understanding how farmers adopt these methods and how they perform across different locations and seasons. Overall, this evidence shows that integrated, environmentally friendly strategies can protect maize, support farmers, and promote resilient, low-input farming systems. Future work should focus on long-term field trials, practical adoption, and socio-economic benefits to make these strategies widely accessible. A systematic literature search was conducted using databases such as Scopus, Web of Science, and Google Scholar covering studies published between 2000 and 2025.

## Introduction

Maize (*Zea mays* L.) remains one of the most important cereal crops worldwide, serving as a foundation for food security, livestock feed, and industrial raw materials (FAO 2023). In many developing regions, particularly sub-Saharan Africa, maize is more than just a crop; it is central to household nutrition, rural livelihoods, and national agricultural economies (Cairns et al. 2021). Despite its significance, maize productivity is continually constrained by a complex range of insect pests that attack the crop from seedling emergence through storage (Abate et al. 2017, Isman 2020).

Among these pests, fall armyworm (*Spodoptera frugiperda*) has become one of the most serious global threats to maize production. Since its introduction into Africa in 2016, the pest has spread rapidly across continents due to its strong migratory ability, high reproductive rate, and wide host range (Goergen et al. 2016, Day et al. 2017). Fall armyworm larvae feed aggressively on maize leaves and reproductive structures, often causing severe defoliation and yield reductions ranging from 20% to more than 50% under heavy infestation (Montezano et al. 2018, Prasanna et al. 2022). Its capacity to adapt to diverse agroecological conditions and develop resistance to commonly used insecticides further complicates management efforts (Sparks and Nauen 2015, Hruska 2019). Stem borers, particularly *Busseola fusca* and *Chilo partellus*, continue to impose significant constraints in tropical maize systems. These pests damage maize by boring into stems and ears, disrupting nutrient transport and predisposing plants to lodging and secondary infections. Yield losses typically range between 10% and 40%, although under unmanaged conditions, damage can be substantially higher (Ndemah et al. 2015, Abate et al. 2017). Their persistence in smallholder farming systems is closely linked to continuous maize cultivation and limited crop diversification (Lenné and Wood 2024).

Maize aphids (*Rhopalosiphum maidis*) represent another important group of pests. Although direct feeding damage is often moderate, aphids weaken plants by extracting sap and, more importantly, transmit viral diseases that can severely affect crop performance. Under favorable conditions, aphid infestations can lead to indirect yield losses approaching 30% due to virus transmission and reduced photosynthetic capacity (Prasanna et al. 2018, Blackman and Eastop 2020). Maize weevil (*Sitophilus zeamais*) remains one of the most destructive post-harvest pests, particularly in regions relying on traditional storage systems. Grain losses during storage may range from 15% to 40%, reducing both food availability and market value (Tefera et al. 2011). Post-harvest infestations not only compromise grain quality but also undermine the economic stability of smallholder farmers who depend on stored maize for income and household consumption (Muwereza 2024).

Historically, synthetic insecticides have been widely adopted because they provide rapid and visible control of pest outbreaks. However, decades of intensive and often unregulated use have resulted in several unintended consequences, including pesticide resistance, pest resurgence, contamination of soil and water resources, and negative effects on beneficial organisms and human health (Sparks and Nauen 2015, Nicolopoulou-Stamati et al. 2016). In many maize-growing regions, improper application practices and limited access to safety training further exacerbate these risks (Pretty and Bharucha 2015).

Growing awareness of these challenges has stimulated renewed interest in environmentally sustainable pest management approaches. Botanical insecticides derived from plants such as *Azadirachta indica* and other locally available species have demonstrated promising bioactivity against a range of maize pests. These plant-based products often act as feeding deterrents, growth regulators, and toxicants, while degrading more rapidly in the environment than conventional synthetic chemicals (Pavela and Benelli 2016, Isman 2020). Their relatively lower toxicity to non-target organisms makes them attractive components of sustainable crop protection strategies. Cultural practices also play a fundamental role in reducing pest pressure. Techniques such as intercropping, crop rotation, optimized planting dates, and field sanitation can disrupt pest life cycles, enhance natural enemy populations, and create less favorable environments for infestation (Abate et al. 2017, Sharma et al. 2020). These approaches are often low-cost and well-suited to smallholder production systems, although their effectiveness depends on local ecological and socio-economic conditions. The aim of this review is to bring together and evaluate existing research on botanical and cultural strategies for managing major insect pests of maize, and to highlight their potential role in developing more sustainable pest management systems.

## Materials and Methods

### Review Design

This study was designed as a systematic literature review to provide a comprehensive and reliable synthesis of existing research on botanical and cultural control methods for maize insect pests. The review process followed recognized PRISMA guidelines for systematic reviews in agricultural and environmental sciences to ensure transparency, consistency, and reproducibility (Liberati et al. 2009). Before beginning the literature search, the scope and objectives of the review were clearly defined. The focus was on evaluating plant-based insecticides, cultural management practices, and integrated approaches used to control major maize pests under field and laboratory conditions.

### Literature Search Strategy

A comprehensive search of scientific literature was conducted using four major academic databases: Web of Science, Scopus, PubMed, and Google Scholar. Peer-reviewed articles published between January 2000 and March 2025 were considered. Greater emphasis was placed on studies published from 2015 onward to capture recent advances in sustainable maize pest management. The search strategy combined relevant keywords using Boolean operators. The main search terms included “maize pests” OR “*Zea mays* L. *pests*,” “fall armyworm” OR “*Spodoptera frugiperda* J.E. Smith,” “stem borers” OR “*Busseola fusca* Fuller” OR “*Chilo partellu* Swinhoe,” “maize aphids” OR “*Rhopalosiphum maidis* Fitch,” “maize weevil” OR “*Sitophilus zeamais* Motschulsky.” These were used together with management-related terms, including “botanical insecticides” OR “plant extracts” OR “biopesticides,” as well as cultural practices such as “intercropping” OR “crop rotation” OR “planting date.” In addition, broader terms like “integrated pest management” OR “IPM.” Only articles published in English and appearing in peer-reviewed journals were included to maintain scientific quality. In addition, reference lists of selected articles were carefully screened to identify other relevant studies that may not have appeared in the initial database search.

### Study Selection Criteria

To ensure relevance and consistency, clear inclusion and exclusion criteria were applied following PRIZMA guidelines. The studies were included if they focused on maize (*Zea mays* L.) production systems, evaluated botanical insecticides, plant-derived extracts, or cultural pest management practices, and reported measurable outcomes such as pest mortality, infestation reduction, population suppression, yield response, or ecological effects. Eligible studies also needed to be conducted under laboratory, greenhouse, field, or farm conditions and published in peer-reviewed scientific journals. The exclusion criteria excluded studies that focused only on synthetic insecticides without comparison to botanical or cultural approaches, investigated crops other than maize, or were not published in peer-reviewed journals. Conference abstracts, technical reports, dissertations, and studies lacking sufficient methodological detail or usable outcome data were also excluded. Before screening began, duplicate records were removed. The selection process then followed a PRISMA-style approach, starting with screening titles and abstracts to remove clearly irrelevant studies, followed by a full-text review of the remaining articles. Studies that did not meet the eligibility criteria at the full-text stage were excluded, and the reasons for exclusion were recorded.

### Data Extraction

Data were extracted using a simple and consistent format to keep everything uniform across the included studies. The first author handled the extraction of all key information from each paper, such as study details, pest management methods, and main outcomes like pest mortality, infestation levels, and yield effects. To make sure nothing was missed or recorded incorrectly, a second and third author independently checked the extracted data. Any differences between the three authors were discussed and agreed upon together. A structured data extraction form was developed to ensure consistency in collecting relevant information from each selected study. The following information was recorded: authors and year of publication, geographic location or agro-ecological zone, experimental design (laboratory, greenhouse, or field), and duration of the study. The pest information included target pest species, life stage evaluated (egg, larva, nymph, adult), and severity or level of infestation. The botanical insecticides and cultural practices that were used included like botanical species used (e.g., *Azadirachta indica*, *Tephrosia vogelii*), plant part utilized (leaf, seed, oil, bark), extraction method (aqueous, ethanolic, oil-based), cultural practice applied (intercropping, crop rotation, planting date adjustment, sanitation), and integrated strategies combining multiple approaches. The outcome measurement was used based on percentage mortality, reduction in pest population or infestation (%), yield improvement (%), effects on non-target organisms, and farmer adoption or socio-economic observations.

### Quality Assessment

To make the review more reliable, we carefully assessed the quality of all included studies. Each study was checked based on how clearly it described its methods and how strong its scientific design was. We looked at factors such as the experimental setup, number of replications, use of statistical analysis, study duration and scale, whether the work was done in the field or laboratory, and how clearly the results were reported. Based on these aspects, studies were grouped as high, moderate, or low quality. Studies with stronger design and clearer reporting were considered more reliable, while those with limited details were treated with more caution during interpretation. This approach follows good practice in systematic agricultural reviews (Haddaway et al. 2018).

### Data Synthesis

Due to variation in study designs, environmental conditions, pest species, and outcome measurements, a qualitative synthesis approach was adopted instead of a formal meta-analysis. Evidence was synthesized narratively by grouping studies with similar outcomes, and trends were identified based on the frequency of reported effects. Outcome ranges were derived from the minimum and maximum values across studies rather than statistical pooling. Greater interpretive weight was given to studies with stronger methodological rigor and consistent findings. The findings were therefore organized into three main thematic categories: (i) botanical control methods, (ii) cultural control practices, and (iii) integrated botanical and cultural strategies. Within each category, trends were identified based on consistency of pest suppression, reported yield responses, ecological safety, and feasibility for smallholder adoption. Particular attention was given to recent research (2015–2025) to reflect current developments in sustainable pest management (Pretty and Bharucha 2015, Isman 2020).

## Results

### Study Selection and General Characteristics

The literature search across Web of Science, Scopus, PubMed, and Google Scholar identified 1,284 records. After removing 312 duplicate articles, 972 studies were screened by title and abstract. From these, 684 were excluded because they did not meet the inclusion criteria (e.g., non-maize crops, synthetic insecticides only, non-peer-reviewed publications, or insufficient outcome data). A total of 288 full-text articles were assessed in detail, and 34 studies met all eligibility requirements and were included in this review following PRISMA guidelines (Page et al. 2021).

Most of the included studies (71%) were published between 2015 and 2025, indicating increasing scientific interest in sustainable maize pest management (Pretty and Bharucha 2015). Nearly half of the studies were conducted in sub-Saharan Africa (46%), followed by Asia (28%) and Latin America (18%). The remaining studies (8%) were from Europe and North America. Field experiments were the most common study design (58%), followed by laboratory bioassays (27%) and greenhouse trials (15%). The most frequently investigated pests were *Spodoptera frugiperda*, *Busseola fusca*, *Chilo partellus*, *Rhopalosiphum maidis*, *and Sitophilus zeamais.* These pests represent both field and storage threats to maize production systems (Harrison et al. 2019).

### Botanical Control Methods

#### Plant Species and Preparation Methods

Across the 34 studies, 23 different plant species were evaluated for insecticidal activity. The most commonly studied botanicals were *Azadirachta indica*, *Tephrosia vogelii*, *Ocimum basilicum*, *Lippia javanica*, and *Eucalyptus globulus* (Table 1). Leaves (44%) and seeds (29%) were the most frequently used plant parts. Most studies prepared extracts using water (52%), followed by ethanol (21%), essential oil distillation (18%), and methanol (9%). This reflects the practical preference for low-cost and locally accessible extraction methods, particularly in smallholder farming systems (Stevenson et al. 2017).

**Table 1.** Performance of major botanical insecticides in maize pest management (2000–2025)

Botanical species	Target pest	Study setting	Pest mortality/reduction (%)	Yield increase (%)	Reported non-target impact	Source
***Azadirachta indica***	*S. frugiperda*	Lab/field	55–92%	18–32%	Minimal	Campos et al. (2015)
***Tephrosia vogelii***	Stem borers	Lab/field	60–85%	15–28%	Low	Mkenda et al. (2015)
***Ocimum basilicum***	Aphids	Laboratory	50–78% (repellency)	Not reported	Safe to pollinators	Pavela and Benelli (2016)
***Lippia javanica***	FAW	Field	35–61%	14–22%	Minimal	Midega et al. (2018)
***Eucalyptus globulus***	Aphids/borers	Laboratory	48–76%	Not reported	Dose-dependent	Nerio et al. (2010)

#### Effectiveness against Maize Pests

Botanical insecticides generally showed promising, though variable, effectiveness depending on the plant species, target pest, and experimental conditions. Several studies reported strong performance of neem-based products, with larval mortality of fall armyworm ranging from 45% to 92% under laboratory conditions (Isman 2020). Similarly, ethanolic extracts of *Tephrosia vogelii* achieved 60–85% mortality against stem borer larvae, while essential oils from *Ocimum basilicum* and *Eucalyptus globulus* provided 50–78% repellency or feeding inhibition against aphids and early-stage larvae (Stevenson et al. 2017). In storage systems, neem seed powders were particularly effective, reducing maize weevil emergence by 70–95%. Field studies, although generally reporting lower efficacy than laboratory trials, still demonstrated consistent benefits, with pest infestations reduced by 30–68% and grain yields increased by 12–34% compared to untreated controls (Mkindi et al. 2017). Overall, while laboratory results tended to show higher mortality rates, field evidence confirms that botanical insecticides can provide meaningful pest control under real farming conditions.

### Cultural Control Practices

#### Intercropping Systems

Intercropping maize with legumes or other companion plants was consistently associated with reduced pest pressure. In particular, push–pull systems using *Desmodium* species as repellent intercrops and Napier grass (*Pennisetum purpureum*) as trap crops showed substantial reductions in pest infestation. Fall armyworm infestation decreased by 40–75%, while stem borer damage was reduced by up to 80% (Khan et al. 2010**)**. These systems also led to yield improvements of 20–45% compared with maize monocropping, especially in smallholder farming systems (Table 2).

**Table 2.** Effectiveness of cultural practices in maize pest suppression

Cultural practice	Target pest	Infestation reduction (%)	Yield response (%)	Feasibility	Source
**Intercropping**	FAW, stem borers	40–75%	20–45%	High	Harrison et al. (2019)
**Crop rotation**	Stem borers, weevils	25–58%	12–30%	Moderate	Baudron et al. (2019)
**Planting date adjustment**	FAW	18–47%	8–21%	High	Early et al. (2018)
**Field sanitation**	Stem borers	30–60%	10–24%	Moderate	Belmain et al. (2001)

#### Crop Rotation and Planting Date Adjustment

Crop rotation with non-host crops was found to reduce pest carry-over populations, with reductions ranging from 25% to 58% for stem borers and maize weevils (Altieri and Nicholls 2017). Adjusting planting dates to avoid peak pest periods also proved effective, reducing fall armyworm infestation by 18–47%, depending on local environmental conditions **(**Harrison et al. 2019**)**. These approaches were generally practical and adaptable within farmer-managed systems.

#### Field Sanitation

Field sanitation practices, such as removing or destroying infested crop residues, contributed to reductions in overwintering stem borer populations by 30–60%. When combined with other cultural practices, these measures further improved overall pest management outcomes (Kfir et al. 2002). For example, when combined with rotation or intercropping, sanitation practices further improved pest suppression.

#### Integrated Botanical and Cultural Strategies

Studies that combined botanical insecticides with cultural practices consistently reported stronger and more reliable outcomes than those using single methods alone (Table 3). For example, neem-based treatments used alongside intercropping reduced fall armyworm damage by 65–88%, while push–pull systems supplemented with botanical sprays increased yields by up to 52% **(**Khan et al. 2010, Mkindi et al. 2017). In storage systems, combining botanical seed treatments with improved sanitation reduced maize weevil damage by more than 90% over a 6-month period. Beyond pest control, integrated approaches also contributed to ecological sustainability. Several studies reported higher populations of beneficial organisms, such as parasitoids and predatory arthropods, in systems using botanical treatments and intercropping compared to those relying on conventional insecticides (Pretty and Bharucha 2015**).** 

**Table 3.** Performance of integrated botanical–cultural approaches

Integrated strategy	Target pest	Pest reduction (%)	Yield increase (%)	Ecological observation	Source
**Neem + intercropping**	Fall armyworm	65–88%	28–52%	Natural enemies preserved	Harrison et al. (2019)
**Push–pull + botanicals**	Stem borers, Fall armyworm	70–85%	35–50%	Increased parasitoid activity	Pickett et al. (2014)
**Botanical storage treatment + sanitation**	Maize weevil	80–95%	Reduced post-harvest loss	Safe for farmers	Stevenson et al. (2017)

## Discussion

### Growing Scientific Interest and Regional Focus

This review brings together evidence from 34 peer-reviewed studies published between 2000 and 2025, with most (71%) appearing after 2015. This clear rise in publications reflects increasing global concern about pesticide resistance, environmental pollution, and food safety issues linked to synthetic insecticides (Sparks and Nauen 2015, Isman 2020). The rapid spread of the *Spodoptera frugiperda* has also played a major role in driving research attention toward more sustainable maize pest management options, especially in Africa and Asia (Harrison et al. 2019, FAO 2023). The strong representation of studies from sub-Saharan Africa reflects both the region’s high vulnerability to maize losses and its reliance on smallholder farming systems (Day et al. 2017, Midega et al. 2018). Overall, this geographic trend aligns well with global calls for more climate-resilient and agroecological pest management approaches (Pretty and Bharucha 2015, Van den Berg et al. 2021). The dominance of field-based studies is particularly important because it reflects real farming conditions, where pest control products often perform differently than in laboratory settings (Pavela and Benelli 2016, Isman 2020).

### Botanical Insecticide Efficacy and Practical Relevance

The evidence shows that botanical insecticides can be effective, although their performance is not always consistent. Plants such as *Azadirachta indica* and *Tephrosia vogelii* stand out because they are widely available and contain well-known insecticidal compounds (Stevenson et al. 2017, Isman 2020). However, one important point is that laboratory results often appear very strong, while field results are more moderate. This difference is mainly due to environmental factors like sunlight, rainfall, and degradation of active compounds, which reduce effectiveness under natural conditions (Pavela and Benelli 2016). This highlights why laboratory success should not be directly interpreted as field performance. Another key limitation is the lack of standardization in how plant extracts are prepared and applied. This makes it difficult to compare results across studies or recommend a single best method. Even so, botanical insecticides still have a major advantage: they are generally safer for beneficial organisms such as pollinators and natural enemies compared to synthetic insecticides (Stevenson et al. 2017, Mordue and Nisbet 2019).

### Cultural Control Practices and Ecological Mechanisms

Cultural pest control methods such as intercropping, crop rotation, planting date adjustment, and field sanitation consistently showed useful pest reduction effects. However, their impact is usually moderate when each method is used alone. For example, intercropping systems such as the push–pull strategy using *Desmodium uncinatum* and *Pennisetum purpureum* work well because they disrupt pest movement and create unfavorable conditions for infestation (Khan et al. 2017, Midega et al. 2018). Still, their success depends heavily on farmers’ knowledge, management practices, and local environmental conditions. Similarly, crop rotation and planting time adjustments help break pest life cycles, but their effectiveness can vary depending on climate and timing (Altieri and Nicholls 2017, Harrison et al. 2019). Field sanitation is also useful, but it requires continuous labor, which may be challenging for smallholder farmers (Van den Berg et al. 2021).

### Integrated Botanical and Cultural Strategies: Evidence of Synergy

One of the strongest messages from this review is that combining botanical and cultural practices leads to better and more stable pest control than using either approach alone. This happens because different control mechanisms work together. Botanical insecticides provide chemical deterrence, while cultural practices disrupt pest ecology and support natural enemies (Bommarco et al. 2013, Khan et al. 2017). As a result, integrated systems tend to be more resilient and effective over time. However, most studies still focus on short-term experiments. There is limited evidence on long-term performance, economic viability, and farmer adoption. These gaps need attention before large-scale implementation can be fully recommended.

### Methodological Quality and Research Gaps

Although most studies used proper experimental designs and statistical analysis, a key gap remains in socio-economic evaluation. Very few studies examined whether farmers can easily adopt these methods, whether they are cost-effective, or how labor-intensive they are (Isman 2020). This is important because even highly effective technologies may fail if they are not practical for farmers. Future research should therefore combine biological effectiveness with economic and social feasibility. Long-term, multi-location studies are also needed to understand how these strategies perform under different climates and farming systems (Pretty and Bharucha 2015, Van den Berg et al. 2021).

### Implications for Sustainable Maize Production

The evidence suggests that botanical insecticides and cultural practices can meaningfully reduce pest pressure and improve maize productivity. However, real field performance is usually lower and more variable than laboratory results, which highlights the importance of testing under real farming conditions. Integrated pest management systems that combine multiple approaches appear to be the most promising pathway forward. These systems reduce dependence on synthetic pesticides, support biodiversity, and strengthen ecosystem services (Bommarco et al. 2013, FAO 2023). To move forward, future research should focus on long-term validation, standardized methods, and practical cost-benefit analysis to support real-world adoption.

## Conclusion

This review shows that plant-based insecticides and simple cultural practices can effectively manage major maize pests, including *Spodoptera frugiperda*, *Busseola fusca*, *Chilo partellus*, *Rhopalosiphum maidis*, and *Sitophilus zeamais*. The reviewed studies showed that plant-based extracts such as *Azadirachta indica*, *Tephrosia vogelii*, and different essential oils were able to reduce pest populations by about 30% to 95%, depending on the specific study and application. In a similar way, cultural practices including intercropping, crop rotation, adjusting planting dates, and field sanitation helped to lower pest infestations and improve yields by up to 45% across the studies. Combining these approaches tended to give the best results in many of the reviewed studies, with some reporting yield gains of over 50% and better protection of beneficial insects. However, the current literature still provides limited information on challenges related to adoption, long-term field validation, and economic assessment. The reviewed studies suggest that promoting integrated botanical and cultural strategies may help reduce dependence on synthetic pesticides, improve ecological resilience, and support stronger maize production among smallholder farmers. Future research should focus on practical, multi-location studies that link agronomic, ecological, and socio-economic benefits to ensure wide adoption.

## Data Availability

The data used to support the findings of this study are available upon request from the corresponding author.
